# Cryptic prophages within a *Streptococcus pyogenes* genotype *emm*4 lineage

**DOI:** 10.1099/mgen.0.000482

**Published:** 2020-11-27

**Authors:** Alex Remmington, Samuel Haywood, Julia Edgar, Luke R. Green, Thushan de Silva, Claire E. Turner

**Affiliations:** ^1^​ Department of Molecular Biology and Biotechnology, Florey Institute, University of Sheffield, Sheffield, UK; ^2^​ Department of Molecular Biology, Princeton University, Princeton, NJ, USA; ^3^​ Department of Infection, Immunity and Cardiovascular Disease, Florey Institute, University of Sheffield, Sheffield, UK

**Keywords:** bacteriophage, DNA mismatch repair, DNase, group A *Streptococcus*, SpyCI, superantigen

## Abstract

The major human pathogen *Streptococcus pyogenes* shares an intimate evolutionary history with mobile genetic elements, which in many cases carry genes encoding bacterial virulence factors. During recent whole-genome sequencing of a longitudinal sample of *S. pyogenes* isolates in England, we identified a lineage within *emm*4 that clustered with the reference genome MEW427. Like MEW427, this lineage was characterized by substantial gene loss within all three prophage regions, compared to MGAS10750 and isolates outside of the MEW427-like lineage. Gene loss primarily affected lysogeny, replicative and regulatory modules, and to a lesser and more variable extent, structural genes. Importantly, prophage-encoded superantigen and DNase genes were retained in all isolates. In isolates where the prophage elements were complete, like MGAS10750, they could be induced experimentally, but not in MEW427-like isolates with degraded prophages. We also found gene loss within the chromosomal island SpyCIM4 of MEW427-like isolates, although surprisingly, the SpyCIM4 element could not be experimentally induced in either MGAS10750-like or MEW427-like isolates. This did not, however, appear to abolish expression of the mismatch repair operon, within which this element resides. The inclusion of further *emm*4 genomes in our analyses ratified our observations and revealed an international *emm*4 lineage characterized by prophage degradation. Intriguingly, the USA population of *emm*4 *S. pyogenes* appeared to constitute predominantly MEW427-like isolates, whereas the UK population comprised both MEW427-like and MGAS10750-like isolates. The degraded and cryptic nature of these elements may have important phenotypic and fitness ramifications for *emm*4 *S. pyogenes *, and the geographical distribution of this lineage raises interesting questions on the population dynamics of the genotype.

## Data Summary

Complete genomes were obtained for four isolates and these were submitted to the National Center for Biotechnology Information (NCBI) under the BioProject number PRJNA660931 and accession numbers are provided in Table S1 (available with the online version of this article). All raw sequence data used in this study has been previously published and was obtained from the NCBI short-read archive. The accession numbers and citations for this genome data for each individual isolate are provided in Table S2.

**Impact Statement**

*
Streptococcus pyogenes* is a highly dynamic human pathogen with the capacity to cause a breadth of self-limiting and potentially devastating clinical syndromes. Toxigenic temperate bacteriophage appear to be the predominant contributories of genetic diversity in *S. pyogenes* both within and between genotypes, and are recurrently identified as catalysts in the emergence of new or unusual variants, marked changes in infection character, and in the triggering of outbreaks. Genotype *emm*4 *S. pyogenes* are polylysogenic and constitute a major source of disease burden globally. Unlike a number of other dominant genotypes, the evolutionary pathways and population dynamics of which have been illuminated with the application of whole-genome sequencing, hitherto, the international *emm*4 population has not been characterized. This report constitutes what is believed to be the first description of prophage inactivation as a trigger for phylogenetic divergence within *S. pyogenes *, and represents a novel mechanism by which mobile genetic elements in this species can shape the evolution of the population that is independent of their initial influence following acquisition. Together, these data may provide valuable insight into the mechanisms by which mobile genetic elements shape the genome of this often highly lysogenized species and, more broadly, may enhance our understanding on the acquisition and domestication of virulence determinates by pathogenic bacteria.

## Introduction

*Streptococcus pyogenes* (also known as the Lancefield group A *Streptococcus* or GAS) is a globally distributed human pathogen [1]. The spectrum of infections caused by *S. pyogenes* is broad, with manifestations ranging from the relatively mild and self-limiting tonsillitis and scarlet fever, to the more severe, invasive and potentially life-threatening, notably necrotizing fasciitis and streptococcal toxic shock syndrome [2]. *S. pyogenes* isolates are classified into *emm* genotypes in accordance with the 5′ hypervariable region of the *emm* gene, which encodes the surface protein M [3, 4].

Most *S. pyogenes* genotypes are lysogenized by at least one prophage, though it is not at all uncommon for certain genotypes to carry several such elements integrated into the bacterial chromosome, or indeed none [5]. As is the case for a number of other bacterial pathogens, the lysogenic bacteriophage of this species often are associated with bacterial virulence factors. Eight of the thirteen superantigens in *S. pyogenes* (*ssa, speA*, *speC*, *speH*, *speI*, *speK*, *speL* and *speM*, and allelic variants thereof) are prophage-associated [6, 7]. In addition, prophage in this species often carry genes encoding a potential six secreted nucleases (*sdn*, *spd1*, *spd3*, *spd4*, *sda* and *sdaD2*, and allelic variants thereof), and a secreted A_2_ phospholipase, denoted *sla* [8, 9]. The *S. pyogenes* chromosomal islands (SpyCIs) are similar but distinct mobile genetic elements, which are more closely related to the *Staphylococcus aureus* pathogenicity islands (SaPIs) than they are to phage [10, 11]. In the *emm*1 isolate SF370, SpyCIM1 is integrated within the DNA mismatch repair (MMR) operon, separating *mutS* from *mutL* and other downstream repair-associated genes, preventing co-transcription of these genes from a promoter upstream of *mutS* [12]. A similar element was also identified in the genomes of isolates belonging to other genotypes, including the *emm*4 MGAS10750 [13]. With the exception of *emm*5 isolate Manfredo, the presence of a SpyCI element within the MMR operon was associated with increased mutation rates, indicating the disruption of the MMR-associated gene transcription [12, 13]. Dynamic integration and excision of SpyCIM1 in SF370 was detected in response to bacterial growth: excising during earlier growth phases to permit transcription of the MMR operon, but remaining integrated at later stages; thus, abolishing transcription and promoting a transient, more mutable phenotype [12, 13]. Intriguingly, curing of SpyCIM1 had a dramatic effect on global transcription, including the expression of a number of well-characterized virulence factors [14]. Together, these studies effectively describe a mechanism of genetic regulation in *S. pyogenes* that is dependent on integration and excision of phage-like elements. There is, however, a general lack of knowledge regarding prophages and phage-like elements and their impact on streptococcal biology, despite their prevalence.

In recent years, whole-genome sequencing (WGS) has provided valuable insight into the biology of this pathogen, illuminating population dynamics and bringing clarity to outbreak investigations and epidemiological shifts. Often, changes in infection character, population structure and disease incidence have been associated with prophage being acquired and/or lost from the population and, with them, cognate bacterial virulence factors, particularly the streptococcal superantigens and DNases [8, 15–21]. Genotype *emm*4 *S. pyogenes* constitute a major *emm*-type in high-income countries globally [1] that is capable of causing both superficial and invasive disease [22–25]. This genotype is genetically acapsular [26], frequently associated with outbreaks of scarlet fever [27–30] and, occasionally, has superseded in incidence the consistently dominant *emm*1 genotype, in some parts of the world [25, 31]. Typically, isolates belonging to this genotype are host to three prophage elements, Φ10750.1, Φ10750.2 and Φ10750.3, encoding the streptococcal superantigen *speC* and the DNase *spd1*, the DNase *spd3*, and another streptococcal superantigen *ssa,* respectively [32–35]. It was recently reported that in an *emm*4 population from Houston, TX, USA, the majority of isolates carried a novel chimeric *emm* gene, formed by the fusion of the 5′ end of *emm*4 with the 3′ end of the downstream gene *enn*. This chimeric *emm*4 gene was also found in the closely related reference *emm*4 genome MEW427, but not the more distantly related MGAS10750 [36].

Despite the prevalence of *emm*4, there has been little genomic work on this genotype so far, particularly regarding prophage gene content. To address this, we explored the genome data of *emm*4 isolates and revealed that within an *emm*4 population from England is a lineage that is characterized by marked gene loss within prophage-encoding regions, although associated superantigen and DNase genes remain intact. This lineage was also identified in a wider international population. The gene loss has rendered the prophages immobile, unable to replicate extra-chromosomally and these are, therefore, cryptic prophages.

## Methods

### Genome sequence analysis of British Society for Antimicrobial Chemotherapy (BSAC) isolates

Short-read Illumina WGS data were obtained for 10 *emm*4 *S. pyogenes* isolates from a collection of 344 *S *. *pyogenes* bloodstream infection isolates submitted to the BSAC from 11 geographical locations within England, and sequenced as part of a previous study [37]. Short-read sequence data were mapped to the reference genome of MGAS10750 using smalt (https://www.sanger.ac.uk/tool/smalt-0/), regions of recombination were predicted, and a phylogenetic tree reconstructed using Gubbins analysis and default parameters [38].

### Prophage/SpyCI gene content

To determine prophage gene presence or absence in the ten BSAC isolates, prophage regions were extracted using *in silico* PCR (https://github.com/simonrharris/in_silico_pcr) from *de novo* assemblies previously performed with a Velvet assembly pipeline [37]. To aid prophage sequence assembly, a second assembly method was performed using SPAdes v3.13.1 (k-mers 21, 33, 55, 77 with --careful) [39] with alignment of contigs to the reference genome MGAS10750 using abacus (http://abacas.sourceforge.net/index.html). For five isolates (BSAC_bs468, BSAC_bs696, BSAC_bs916, BSAC_bs1052 and BSAC_bs1349), full sequences of each of the three prophage regions could be extracted, as they had each assembled within single contigs using the second assembly method. The original Velvet *de novo* assemblies produced similar results, but with some gaps within contigs. Annotation was performed using Prokka and prophage regions visually assessed using Artemis [40]. For the five remaining isolates, the presence of additional prophages with some homology to the three prophages of interest introduced contig breaks within prophage regions. Four of these were then subject to Nanopore long-read sequencing to resolve the prophage regions and complete the genomes. To determine the presence of any further prophages within the ten BSAC isolate genomes, Roary [41] (with 95 % minimum identity) was used to calculate the pangenome and identify accessory genes.

SpyCIM4 assembled within a single contig for all ten BSAC isolates due to its relatively short sequence, and lack of homology to prophages. SpyCI sequences were compared to each other and to the reference genomes of MGAS10750 and MEW427.

### Nanopore sequencing

Long-read Nanopore sequencing was performed on four isolates: BSAC_bs192, BSAC_bs472, BSAC_bs1388 and BSAC_bs1802. DNA was prepared for sequencing using the Oxford Nanopore Technologies (ONT) ligation sequencing kit (SQK-LSK109), according to the manufacturer’s protocols. Four separate whole-genome sequences and a blank negative control were barcoded using the Native Barcoding Expansion 1–12 (EXP-NBD104) and pooled to produce bacterial libraries. Libraries were sequenced with the ONT MinION Mk1C using rev C 9.4 flow cells, and run for 24 h. Resulting trace files were analysed with the ONT Guppy basecaller (version 3.2.10) using high-accuracy basecalling.

Assemblies were performed using Unicycler v0.4.7 [42], combining the fastq Nanopore data with the short-read Illumina data for each isolate. Single contigs were obtained from the genome data of each of the four isolates (Table S1), with 34–75× coverage. Prophage regions were predicted using phaster [43], and curated by visual assessment and blast alignment. Complete genomes were submitted to the NCBI (the accession numbers are shown in Table S1) and annotated with the NCBI Prokaryotic Annotation Pipeline. BSAC_bs400 was not subjected to long-read sequencing, but comparison with the newly completed genome of BSAC_bs1388 allowed for manual determination of Φ10750.1 genes from the additional prophage; Φ10750.2 and Φ10750.3 did assemble within single contigs from the short-read sequence data for this isolate.

### Genome sequence analysis of international *emm*4 isolates

To contextualize our BSAC isolates, publicly available genome sequence data were obtained for *emm*4 from other studies, which had also been used in a previous study [37] (Table S2). This comprised data from USA isolates collected by Active Bacterial Core Surveillance (ABCs) USA in 2015 from invasive infections (*n*=48) [44], Canadian isolates (*n*=8 isolated in 2013 or on an unknown date) [45, 46], isolates collected from across England and Wales by Public Health England (PHE) in 2014/2015 from invasive and non-invasive infections (*n*=153) [27, 47], and Cambridgeshire (UK) isolates collected 2008–2012, also from non-invasive and invasive infections (*n*=4) [48]. These were selected as major studies with available *emm*4 sequence data, and were previously assembled and processed for sequence quality, adapter contamination and assembly quality [37]. We chose to focus on sequence type (ST)39 isolates (or closely related STs differing by a single locus) and excluded those with highly diverse STs (differing by two or more loci) as they represent diverse genetic backgrounds and, therefore, do not reflect the population described here. Short-read sequence data were mapped to the reference genome of isolate MGAS10750 using smalt. Prophage and SpyCI regions were removed from the alignment (positions in MGAS10750 : 54 6 031–589 487, 804 157–841 762, 1 220 848–1 256 149, 1 862 867–1 876 370), and core SNPs were extracted by SNP-sites [49] and used to generate a maximum-likelihood phylogenetic tree using RAxML v8.2.12 [50] with the GTR substitution model and 100 bootstraps. *De novo* assembly had already been performed on the genome sequence data for these isolates using Velvet [37]. Prophage gene presence/absence was detected by extracting the DNA sequence of each of the annotated coding regions within each prophage in MGAS10750 to blast the *de novo* assemblies. Gene presence was assigned with ≥99 % match over 100 % length. SpyCIM4 regions, including the flanking *mutL* and *mutS* genes, were extracted from the *de novo* assemblies using *in silico* PCR as, in all cases, this region had assembled across a single contig. In the absence of a SpyCIM4 element, only the sequence for the *mutL/mutS* locus was obtained by this *in silico* PCR method.

### Figure annotation

Figures comparing prophages and SpyCI regions were constructed using EasyFig [51]. Genes were classified according to blast into eight categories: integrase, regulation, hyaluronidase, replication, infection, structural, virulence and, where present, transposon. A ninth hypothetical/indeterminate category was assigned to proteins returning a hypothetical protein result, or identities that did not yield sufficient information to definitively determine function.

### Prophage induction

The ten BSAC *emm*4 *S. pyogenes* isolates were grown on Columbia agar supplemented with 5 % defibrinated horse blood, then colonies transferred for liquid culture statically in Todd–Hewitt broth (Oxoid) at 37 °C, supplemented with 5 % CO_2_. Overnight liquid cultures were diluted 1 : 10 and grown to OD_600_ 0.3 before cultures were split into two 50 ml aliquots. One aliquot was treated with 0.2 µg mitomycin C ml^−1^ (Sigma-Aldrich) and the second served as an untreated control. Cultures were grown for an additional 3 h before the culture was pelleted by centrifugation and DNA was extracted. Bacterial DNA was extracted using the method of Pospiech and Neumann [52]. Isolates were also cultured exponentially from 1 : 10 dilutions of overnight cultures, and DNA extracted from aliquots at given time points. To detect excision and integration of each of the three prophages and SpyCIM4, specific sets of four primer combinations were designed (Table S3). PCR was performed using each primer combination.

### RNA extraction and transcription detection

RNA was extracted from one M4_complete_ isolate and one M4_degraded_ isolate cultured exponentially for 3 h using a using a hot acidic phenol method, as described previously [7]. RNA samples were then DNase treated with Turbo DNase-*free* (Ambion) for 30 min at 37 °C, and 5 µg was converted to cDNA by reverse transcription using Transcriptor reverse transcriptase (Roche) and random hexaoligos (Sigma-Aldrich). A corresponding RT-negative reaction was also performed for each sample, whereby the reverse transcriptase was excluded as a control for contaminating genomic DNA. Transcription of *mutL* and *mutS* was detected using standard PCR with 100 ng cDNA (or RT-negative equivalent) and primers designed to detect each transcript individually (Table S3).

## Results

### Gene loss within each of three prophage regions

**Fig. 1.**
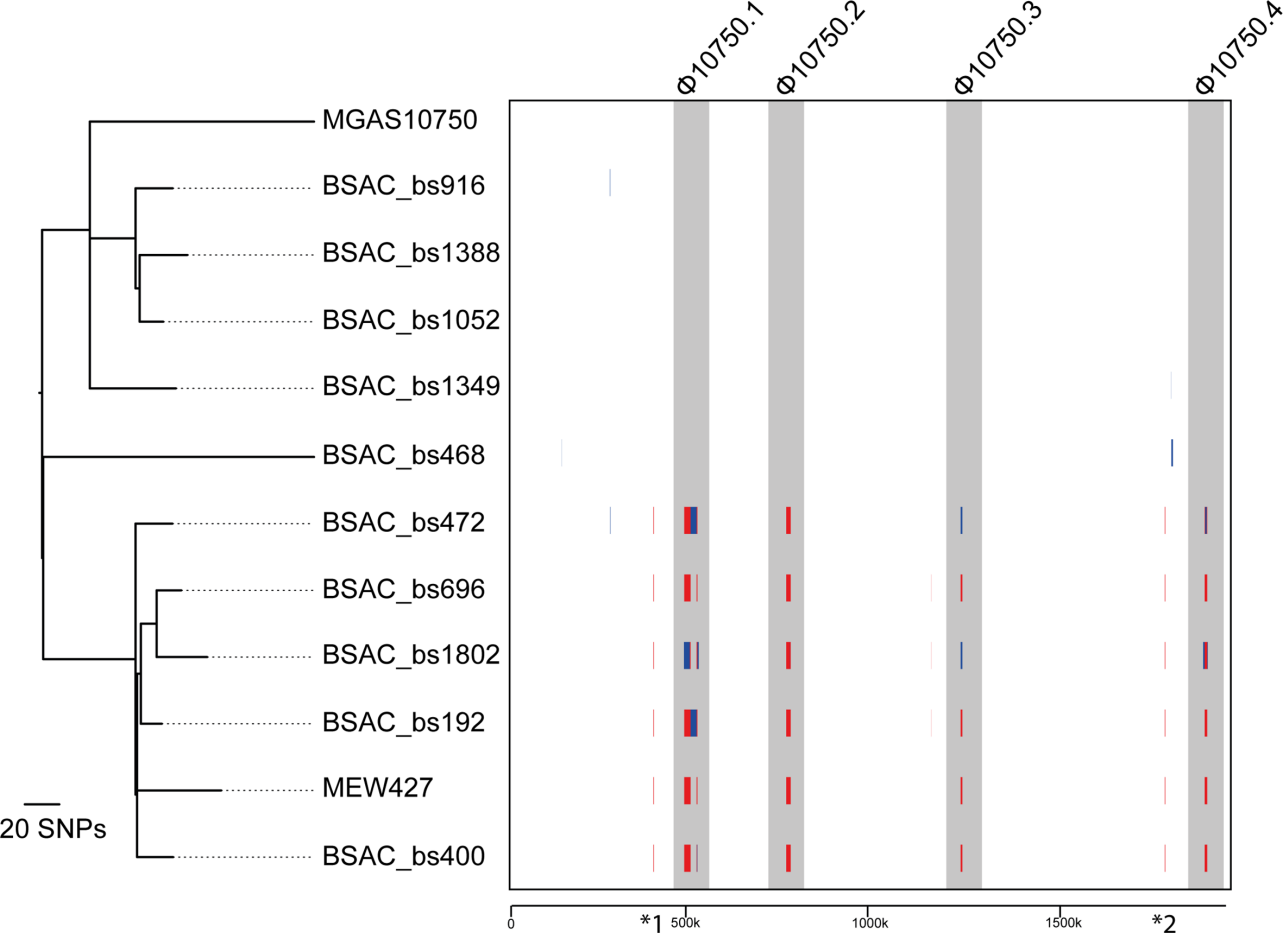
Recombination in prophage regions of isolates clustering with MEW427. Sequence data for all ten BSAC isolates were mapped to the reference isolate MGAS10750 along with a second reference isolate MEW427, and Gubbins analysis performed on the resulting alignment. Vertical red lines indicate recombination common to other isolates (internal nodes) and vertical blue lines indicate recombination on terminal branches. Six predicted regions of recombination were common to all five BSAC isolates and MEW427, which form a separate lineage to the other five BSAC isolates and MGAS10750. Four of these regions were within the three prophages (Φ10705.1, Φ10705.2, Φ10705.3) and the SpyCI element (Φ10705.4) (shaded grey). Two other regions of recombination were also identified within all MEW427-like isolates: *1, relating to the absence of MGAS10750 genes Spy485-488 in MEW427-like isolates; and *2, corresponding to the previously identified chimeric *emm-enn* gene found in MEW427-like isolates [36]. The phylogenetic tree was generated by Gubbins from 556 polymorphic sites following removal of regions of predicted recombination. The tree is mid-point rooted. The scale below boxed region represents genomic position relative to MGAS10750.

We recently undertook WGS of 344 invasive *S. pyogenes* isolates, collected by the BSAC from across England during 2001–2011, and identified 10 *emm*4 isolates within this collection [37]. Within this small population of *emm*4 was a lineage of five isolates that were genetically related to the reference isolate MEW427 [35]. Four of the remaining five isolates were more closely related to the reference isolate MGAS10750 [53] (Fig. 1). A total of 556 core SNPs, outside the regions of predicted recombination, were identified in the population; 34 of these being found in all MEW427-like isolates compared to all MGAS10750-like isolates, differentiating the lineage. Gubbins analysis identified regions of predicted recombination, common to the lineage containing MEW427 and five BSAC MEW427-like isolates, but absent in MGAS10750 and the other five BSAC isolates. Four of the regions of predicted recombination were within each of the three previously identified [53] prophages (Φ10750.1, Φ10750.2, Φ10750.3) and a chromosomal island, SpyCIM4 (Φ10750.4) (Fig. 1).

**Fig. 2.**
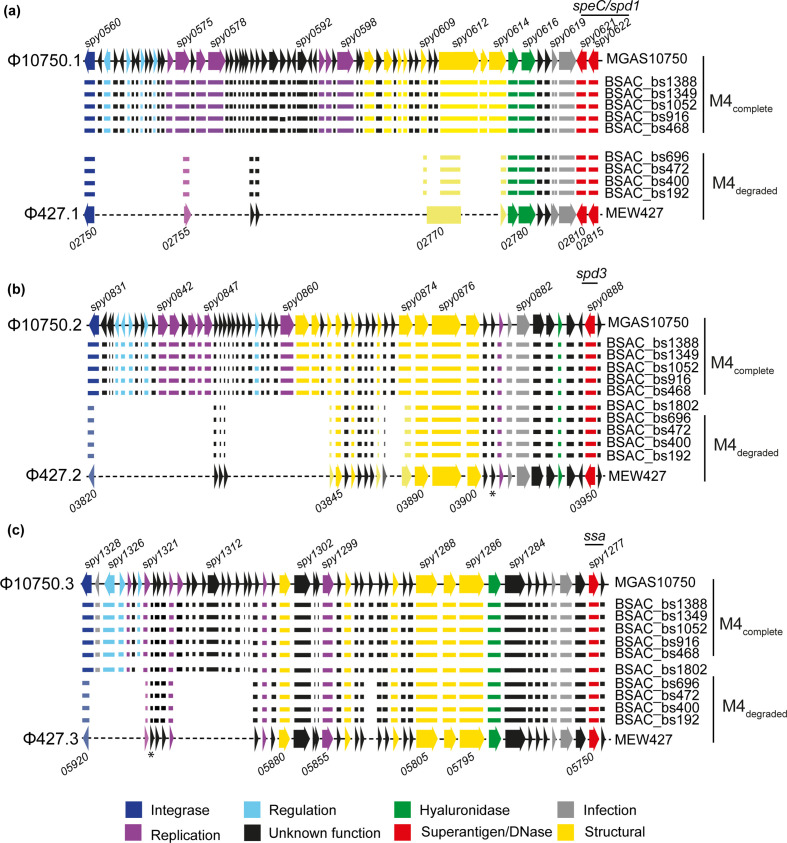
Gene loss within prophage regions. All genes (represented by arrows) present in the three prophages (a) Φ10705.1, (b) Φ10705.2 and (c) Φ10705.3 of MGAS10750 were also present in the five BSAC isolates BSAC_bs468, BSAC_bs916, BSAC_bs1052, BSAC_bs1379 and BSAC_bs1388 (M4_complete_). However, fewer genes were present in the corresponding prophage regions (a) Φ427.1, (b) Φ427.2 and (c) Φ427.3 in MEW427 and the MEW427-like BSAC isolates BSAC_bs192, BSAC_bs400, BSAC_bs472 and BSAC_bs696 (M4_degraded_). BSAC_bs1802 carried a different prophage associated with *speC*/*spd1* and so is not shown in (a). Gene presence in each isolate is represented by corresponding horizontal lines. Some genes in MEW427 and M4_degraded_ isolates were truncated (shown in lighter colours), for example the integrase genes 03820 of Φ427.2 and 05920 of Φ427.3 (lighter shade of blue and shorter arrow/line). Within Φ427.1, the annotated gene *02770* actually comprised sections of *spy609*, *spy0612* and *spy614* of MGAS10750. In all isolate genomes and in MEW427, *spy0619* of Φ10705.1 was divided into two genes. Locus numbers for some genes are provided above and below as a guide; MEW427 is annotated in increments of five. The asterisks indicate an additional gene that was present but not included in the reference annotation. Colours represent predicted gene function according to the key.

Comparison of the three prophage regions between the two completed reference isolates, MGAS10750 and MEW427, actually identified a varying level of sequence and gene loss within these regions in the MEW427 genome relative to the MGAS10750 genome (Figs 2 and S2). To determine whether an equivalent gene loss had occurred in the genomes of the BSAC isolates, we analysed the *de novo* assemblies of the genome sequence data. For five isolates (BSAC_bs468, BSAC_bs696, BSAC_bs916, BSAC_bs1052 and BSAC_bs1349), we could extract the entire sequence of each of all three prophage regions from the *de novo* assemblies as they were within single contigs. The presence of additional prophages within the other five isolates interfered with the *de novo* assembly due to homology between prophages and introduced contig breaks. Four of these isolates (BSAC_bs192, BSAC_bs472, BSAC_bs1388, BSAC_bs1802) were, therefore, subjected to long-read sequencing to resolve the prophage regions and complete genomes were obtained (Table S1). These newly completed genomes were then used to resolve prophage composition within the remaining isolate, BSAC_bs400, which shared a similar additional prophage to BSAC_bs1388. In all isolates, the superantigen and DNase genes associated with each of the prophages were present, and shared 100 % DNA identity between all ten BSAC isolates and the reference strains. The exception was *speC,* wherein a histidine residue was replaced by an asparagine residue in all isolates and MEW427 compared to MGAS10750.

In MGAS10750, the prophage associated with *speC/spd1,* Φ10750.1, was integrated between *whiA* (MGAS10750_Spy0559) and *pepD* (MGAS10750_Spy0623) [53] and comprised 63 genes over ~43.5 kb. In the reference genome MEW427, the corresponding element, Φ427.1, was integrated at the same position. Φ427.1 comprised just 14 genes over ~13.5 kb, equivalent to ~78 % gene loss and ~69 % sequence loss, but with no additional genetic content (Fig. S1). In the *emm*4 isolates in our collection that were associated with the MEW427-like lineage, with the exception of BSAC_bs1802, we found the same pattern of gene presence/absence (Fig. 2a). All five of the other BSAC isolates had all the genes that comprise Φ10750.1, indicating this prophage was complete in these isolates (Fig. 2a).

We did not detect any non-Φ10750.1 prophage genes or sequence within this region in the other four MEW427-like isolate genomes, but we did identify the genes found in Φ427.1 that were altered by the gene loss; MEW427-02755 which is the truncated equivalent of MGAS10750-Spy0575, and MEW247-02770, which is a fusion of regions of MGAS10750-Spy609*,* Spy612 and Spy614 (Fig. 2a). Genes predicted to be involved in regulation and replication of the prophage element were absent in Φ427.1, and in the four MEW427-like BSAC isolates. All predicted structural genes were also absent from the genomes of these isolates, suggesting this prophage would be unable to form bacteriophage particles.

The exception was BSAC_bs1802, which, despite clustering by core genome SNP analysis within the MEW427-like lineage (Fig. 1), in fact carried *speC/spd1* on a different prophage to the rest of the *emm*4 population (Φ1802.1). This prophage was most closely related to a *speC/spd1* prophage found in the genome of *emm*87 isolate NGAS743 [46] (Fig. S2), but did also share regions of homology to Φ10750.1. The integration site remained the same as in MGAS10750/MEW427. As structural genes and other genes predicted to have replication and regulatory roles were present, it seems likely that this would be a full-length lysogenic phage, able to form bacteriophage particles.

The *spd*3-associated prophage Φ10750.2, integrated between MGAS10750_Spy0830 (*cpsFQ*) and MGAS10750_Spy0890 [53], comprised 59 genes over ~37 kb in MGAS10750, and the same was observed in the five MGAS10750-like BSAC isolates (Fig. 2b). However, this element in MEW427 and corresponding elements in all five MEW427-like BSAC isolates, including BSAC_bs1802, comprised only 29 genes over ~20 kb, a loss of ~50 % of genes and ~46 % sequence. This included loss of regulation and replication elements and some structural genes. There were no non-Φ10750.2 genes or sequence present in this region in the MEW427-like isolates but, as in Φ427.1, the sequence loss introduced gene truncations. These included the integrase gene for which the first 184/1140 bases were missing, likely to render the gene non-functional, and we predicted that this prophage would be unable to excise from the chromosome or integrate.

The *ssa*-associated prophage Φ427.3 demonstrated the lesser degree of gene loss compared to Φ10750.3 (~26 %), maintaining the majority of structural genes, although genes predicted to be involved in regulation, and some predicted to be involved in replication, were absent in MEW427 and MEW427-like BSAC isolates, except for BSAC_bs1802 (Fig. 2c). Φ10750.3 is integrated between MGAS10750_Spy1275 and MGAS10750_Spy1329 in MGAS10750 [53], and in the same equivalent position in MEW427. BSAC_bs1802 carried the same *ssa-*encoding prophage as MGAS10750*,* except for one gene, MGAS10750_Spy1322, which in BSAC_bs1802 was replaced with a gene identical to one found in the BSAC_bs1802 *speC/spd1* Φ1802.1 phage; the adjacent gene MGAS10750_Spy1321 was also identical to adjacent genes in BSAC_bs1802 at both sites, so there may have been some homologous recombination between phages in BSAC_bs1802. The other four MEW427-like BSAC isolates had the same pattern of gene presence/absence to MEW427. The integrase gene was also truncated in MEW427 and the four MEW427-like BSAC isolates, with the loss of the first 548/1143 bp. Therefore, we predict that this prophage would also be unable to excise from the chromosome or to reintegrate.

Overall, five BSAC isolates had 100 % of all three prophage genes with limited sequence variation between them (pairwise comparison ≤6 SNPs) and were, therefore, denoted M4_complete_ type, like MGAS10750 (as indicated in Fig. 2). Four BSAC isolates had the same number of genes in Φ10750.1, Φ10750.2 and Φ10750.3, as MEW427, also with limited sequence variation between them (pairwise comparison ≤3 SNPs), and were, therefore, denoted M4_degraded_. In no instance did we identify any novel non-MGAS10750 sequence within the prophage regions, supporting the hypothesis that this was gene loss. BSAC_bs1802 had a different phage associated with *speC/spd1* that was predicted to be complete*,* and a near-complete Φ10750.3. However, a pattern of gene loss that was identical to M4_degraded_ isolates was identified in Φ10750.2 and, thus, was regarded as only partially degraded.

### Prophage excision potential is abolished in isolates with degraded prophage

**Fig. 3.**
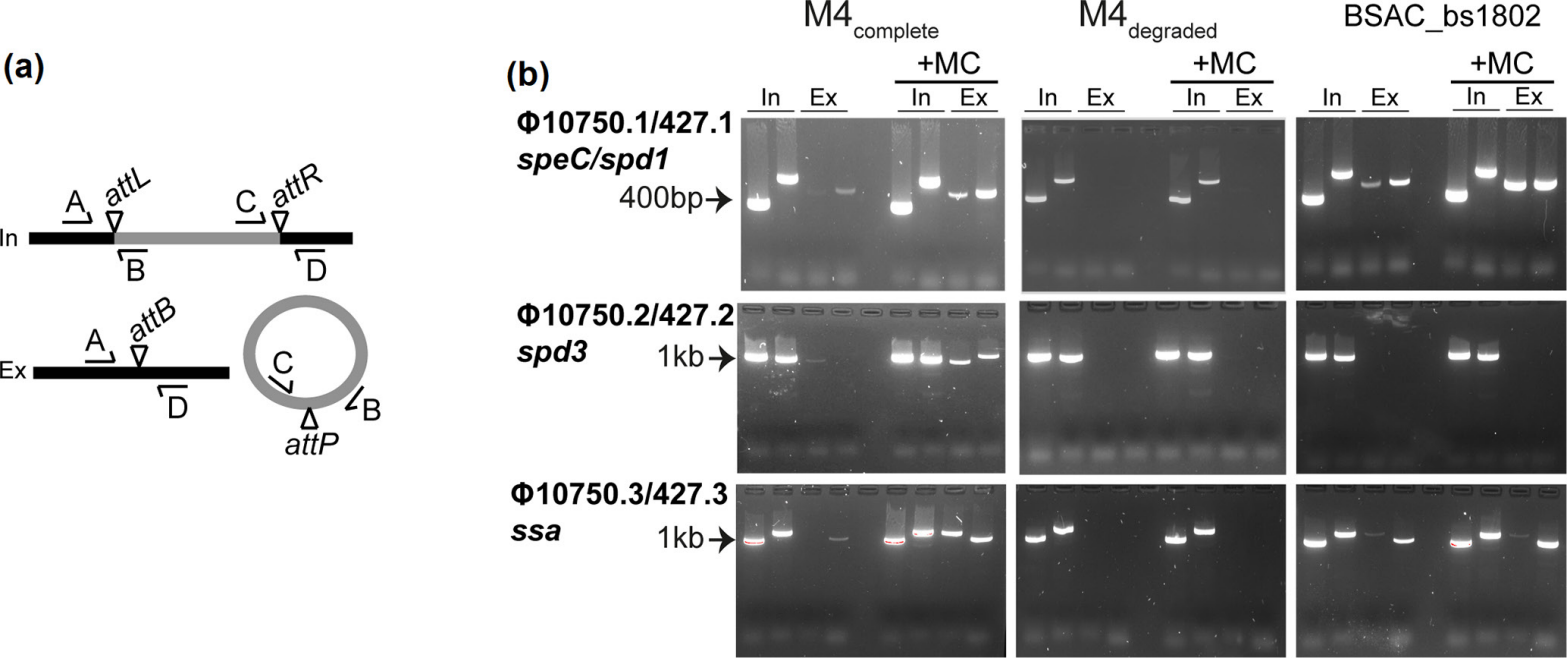
Phage induction in M4_complete_ isolates but not M4_degraded_ isolates. (a) Primers were designed to detect integrated (In) and excised (Ex) prophage. Primer pairs A+B and C+D spanned the attachment sites *attL* and *attR*, respectively, formed when the prophage (grey) is integrated into the chromosome (black). Primer pairs A+D and C+B spanned the attachment site *attB* on the bacterial chromosome and *attP* on the prophage, respectively, detecting prophage excision. (b) Both integrated prophage, as indicated by bands in the two ‘In’ lanes, and excised prophage, as indicated by bands in the two ‘Ex’ lanes (primer pairs A+D and C+B) were detected in M4_complete_ for all three prophages. Excision was enhanced by the addition of mitomycin C (+MC). No excision was detected for all three prophages in M4_degraded_, as indicated by bands only in the In lanes but not the Ex lanes, even with mitomycin C. The exception was BSAC_bs1802, where excision of Φ10750.1/427.1 and Φ10750.3/427.3 was detected but excision of Φ10750.2/427.2 was not. Representative gels are shown for single isolates out of the five M4_complete_ BSAC isolates tested (BSAC_bs1388) and four M4_degraded_ tested (BSAC_bs472). Primer pairs were used in the following order: In lane 1, A+B (*attL*); In lane 2, C+D (*attR*); Ex lane 3, A+D (*attB*); Ex lane 4, C+B (*attP*).

In view of the substantive loss of genes within Φ427.1 and the predicted non-functional integrase genes of Φ427.2 and Φ427.3, we hypothesized that the prophages of M4_degraded_ BSAC isolates would be unable to excise from the chromosome. Using PCR to detect the integrated prophage genome as well as excised and circularized prophage, we found that all three prophage elements spontaneously excise from the chromosomes of M4_complete_ BSAC isolates, and this excision is enhanced with the addition of mitomycin C (Figs 3 and S3). In contrast, we were unable to detect excision of the three degraded prophages from the chromosomes of M4_degraded_ type BSAC isolates, even upon addition of mitomycin C. For the partially degraded BSAC_bs1802, clear excision of the *speC/spd1*-associated prophage was detected as was excision of the *ssa-*associated Φ10750.3/427.3, but not the *spd3*-associated Φ10750.2/427.2. This was expected, as the integrase of Φ10750.2/427.2 was truncated in BSAC_bs1802, like the other MEW427-like isolates.

### Gene loss within the SpyCI element

**Fig. 4.**
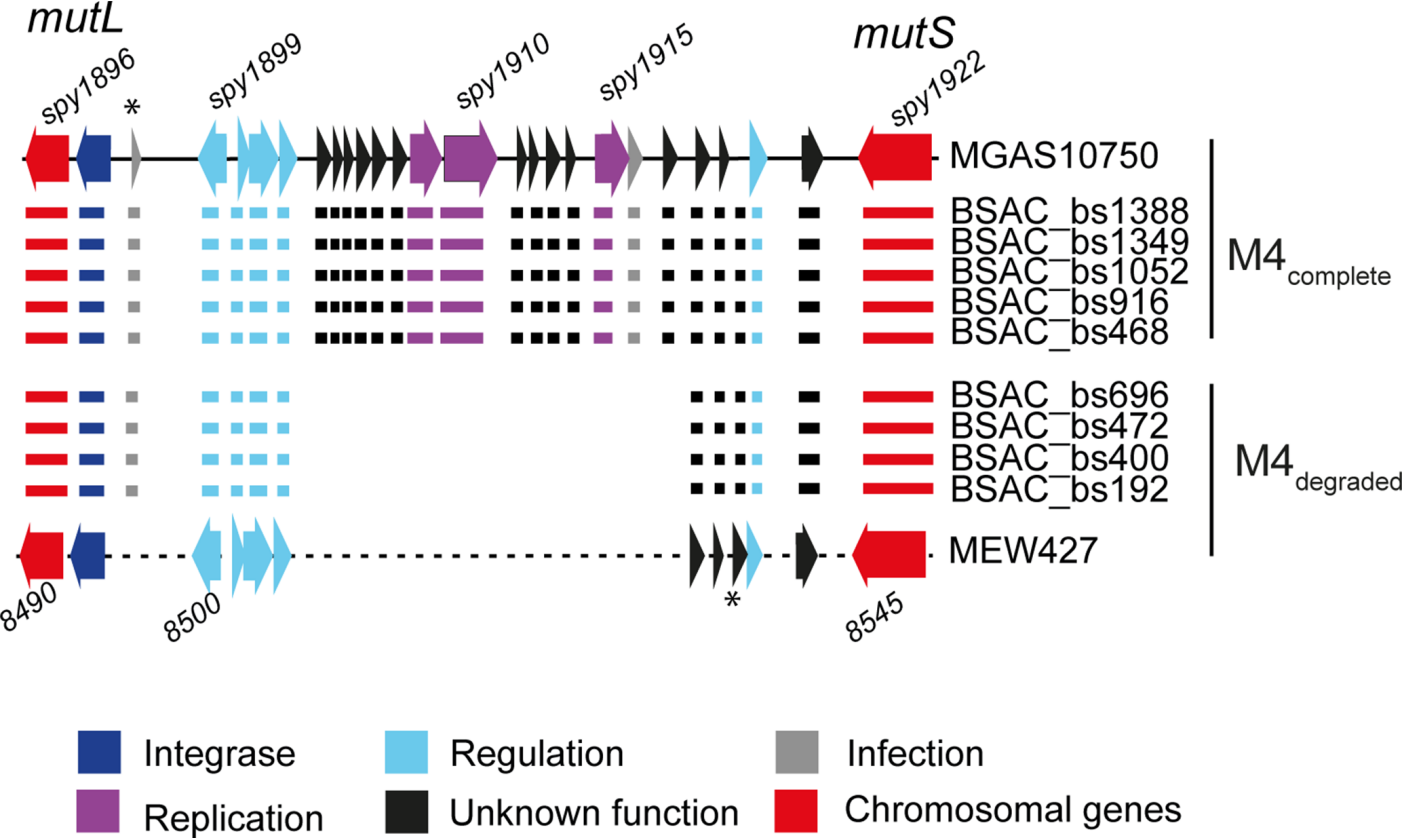
Gene loss within the SpyCI. The SpyCIM4 element is integrated within the MMR operon, between *mutL* and *mutS* (red arrows), and comprises 25 genes at full-length (as in MGAS10750) in the genomes of M4_complete_ isolates. In the genomes of M4_degraded_ isolates, including MEW427, the SpyCI element consists of only 11 genes, representing ~56 % gene loss. The pattern of degradation was common to all M4_degraded_. The SpyCI of BSAC_bs1802 is not shown, as this isolate contains a different SpyCI. Locus numbers for some genes are provided above and below; MEW427 is annotated in increments of five. The asterisks indicate an additional gene that was present but not included in the reference annotation. Colours represent predicted gene function according to the key.

In our five BSAC M4_complete_ isolates, the SpyCIM4 element was identical to that found in MGAS10750, integrated between *mutS* and *mutL*. However, this element shared 97 % DNA identity over just 55 % of the length of SpyCIM1 in SF370. Interestingly, like the prophage elements, MEW427 exhibited some gene loss within the equivalent SpyCI region compared to MGAS10750 (Fig. 4). An identical pattern of gene loss was identified in four of the M4_degraded_ BSAC *emm*4 isolates. The exception was BSAC_bs1802, which appeared to carry a different SpyCI element (Fig. S4) that shared ~99 % identity with a SpyCI found in *emm*28 isolates (e.g. MGAS6180 GenBank accession no. CP000056.2), and *emm*77 isolate NCTC13742 (GenBank accession no. LS483386.1). The SpyCI integrase gene in all *emm*4 isolates was identical. PCR was used to detect excision of SpyCIM4 in M4_complete_ and M4_degraded_ isolates, similar to the three prophage regions. We could not detect excision of this element in M4_complete_ nor M4_degraded_ isolates, even in the presence of mitomycin C (Fig. S5a). Previous studies have shown that this element excises during early exponential growth [12]. Therefore, we tested during exponential growth of one M4_complete_ isolate, but still could not detect excision (Fig. S5b). This was surprising, as when integrated, the element should interrupt the *mutS* and *mutL* operon, preventing the expression of *mutL*. To confirm the lack of expression of *mutL,* we extracted RNA from samples cultured for 3 h, converted the RNA to cDNA, and performed semi-quantitative PCR to detect *mutS* and *mutL* transcription. In all samples, we detected transcripts of both *mutS* and *mutL,* suggesting that despite the consistent integration of SpyCIM4, transcription of *mutL* could still occur (Fig. S6).

### Other prophages within the BSAC isolate genomes

Our short-read sequence assembly and pangenome analysis of the BSAC isolates indicated the presence of one additional prophage within each of five isolates: three M4_degraded_, one M4_complete_ and BSAC_bs1802. Long-read sequencing of four of these isolates allowed us to determine the exact composition of these prophages. BSAC_bs1802 carried a different *speC*/*spd1* prophage (Φ1802.1) to the other *emm*4 isolates and, additionally, carried another prophage just 12 genes downstream of Φ1802.1, which was associated with the DNase *sdn* (Φ1802.2). Φ1802.2 was almost identical to Φ743.3, an *sdn*-encoding prophage in the genome of *emm*87 isolate NGAS743 (CP007560.1), (Fig. S7a), integrated between *gph* (DI45_06350 of NGAS743) and *rplS* (DI45_06680 of NGAS743).

The additional prophage found in M4_degraded_ BSAC_bs192 (Φ192.1) was associated with the superantigen *speK* and the phospholipase *sla* (Fig. S7b). This prophage was integrated between *yesN* and *msrA*; equivalent to MGAS10750_Spy1388 and MGAS10750_Spy1389. A prophage integrated into the same site was also identified in M4_degraded_ BSAC_bs472 (Φ472.1), but lacked *speK/sla*. A comparison between these two prophages identified a high level of homology across the prophage, except for a region of five genes in Φ192.1. This region of Φ192.1 included *speK/sla,* but comprised seven different genes of unknown function in the otherwise similar Φ472.1 (Fig. S7b). An almost identical prophage to Φ472.1 was also found in BSAC_bs1388 (Φ1388.1), but was integrated into a different site, equivalent to MGAS10750_Spy0673 (*pheT*) and MGAS10750_Spy0674. These three prophages, Φ192.1, Φ472.1 and Φ1388.1, shared the closest homology to MGAS29284 as determined by blast against the entire NCBI database, with 98 % identity over 87 % of the length (Fig. S7b). Analysis of BSAC_bs400, which was not subjected to long-read sequencing, also revealed an additional prophage, integrated in the same site as Φ1388.1 and shared 68 % of the Φ1388.1 genes, as determined by blast. Structural, replication, regulation and integrase genes were present in all four homologous prophages, suggesting they could excise and form functional bacteriophages.

### Prophage-associated gene loss is found in other international *emm*4 isolates

**Fig. 5.**
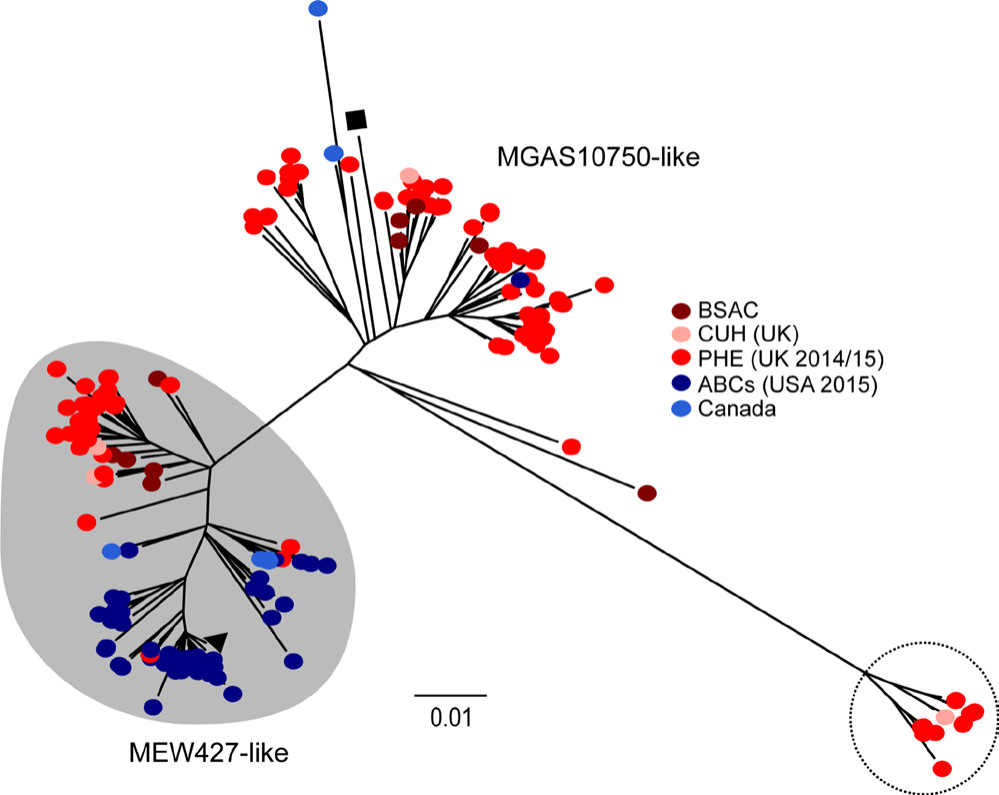
Lineages within the *emm*4 population. Genome sequence data from 223 isolates were obtained and mapped against the reference genome MGAS10750 (black square) and 4994 concatenated core SNPs (excluding prophage and SpyCI regions) used to generate a maximum-likelihood phylogenetic tree. The second reference isolate MEW427 was also included (black triangle). Data comprised genomes from ABCs (USA) isolates (blue circles, *n*=48) [44], Canadian isolates (light blue circles, *n*=8) [45, 46], PHE (UK) isolates (red circles, *n*=153) [27, 47], as well as isolates from Cambridgeshire (CUH, UK) (pink circles, *n*=4) [48] and BSAC isolates (brown circles, *n*=10). Isolates in red and dark blue were obtained around a similar time period (2014–2015), but from either the UK (red) or the USA (blue). Two broad lineages exist, either associated with MEW427 (shaded grey) or MGAS10750 (unshaded). A small lineage was also identified (dotted line circle) and these isolates are all from the UK. Bar, number of substitutions per site.

To place our isolates within the context of a wider *emm*4 population, we obtained publicly available *emm*4 WGS data for isolates from North America and the UK (*n*=223) (Table S2). Phylogenetic analysis identified two broad lineages associated either with MGAS10750 or with MEW427, as well as a third smaller lineage (Fig. 5). As we could not obtain full sequence of the prophages over single contigs for all isolates, prophage gene presence was determined when a ≥99 % match over the full gene length was obtained from blast alignment of each gene within Φ10750.1, Φ10750.2 and Φ10750.3 against the short-read *de novo* assemblies of these isolates. This estimated gene presence/absence to a varying degree (Fig. S8, Table S2). However, there was an association of fewer prophage genes across all three prophages within the lineage clustering with MEW427 when compared to the other lineages. A consistent feature within MEW427, M4_degraded_ and partially degraded BSAC_bs1802 was a deletion of the 5′ region of the integrase gene in the *spd3* phage Φ10750.2/427.2. All 117 genomes of isolates clustering with MEW427 (shaded region in Fig. 5, Table S2) had the same deletion within the integrase gene, like MEW427. In contrast, all of the 106 genomes of isolates outside this lineage had a full-length integrase gene, like MGAS10750. We also found within MEW427 and our M4_degraded_ isolates, a deletion of the 5′ end of the *ssa*-associated prophage integrase. This deletion was found in 112/117 genomes of isolates that clustered with MEW427 (shaded region in Fig. 5) and complete in the remaining five isolates, as well as all isolates outside of this lineage. Taken together, this suggested that the *spd3-* and *ssa*-associated prophages within the majority of isolates clustering within the MEW427-like lineage would not be able to excise from the chromosome and are, therefore, cryptic. We also performed blast alignment of the *de novo* assemblies with the sequence of the MEW427 gene, *02770,* which was unique to the degraded Φ427.1 prophage as it is formed following gene/sequence loss. This was present in 106/117 of the isolates that clustered with MEW427 (Table S2), suggesting that in these isolates Φ10750.1 had undergone degradation similarly to MEW427, and may also be cryptic.

There was a third lineage within the population, comprising 13 UK isolates, for which very few Φ10750.1 genes could be detected, although *speC* and *spd1* genes were present. Prophages Φ10750.2 and Φ10750.3 appeared to be complete, with full-length integrases. Although we could not fully confirm this with the short-read sequence data, blast alignment indicated that these isolates carried Φ1802.1. We did not find any other isolates within the population that carried this prophage.

Two isolates, GASEMM0570 and PHE_45828, had only 6 % of the Φ10750.1 genes, but clustered with MGAS10750 and carried *speC/spd1*. Although we could not determine the exact composition, it did appear that they both carried a different prophage that was not related to Φ10750.1 or Φ1802.1. Additionally, within the whole population, we also identified two isolates that did not carry the *speC* and *spd1* genes or an associated prophage (Table S2).

We also determined the sequence of the SpyCI region in all 223 isolates. This region did assemble within single contigs and so the sequence could be compared between all isolates. BSAC_bs1802 was the only isolate to carry the *emm*28/*emm*77-like SpyCI. The 13 isolates belonging to the third smaller lineage all carried a SpyCIM4 that was different to any variant carried by any other *emm*4 isolate, and did not share any appreciable homology to anything on the NCBI database (Fig. S4). Across all lineages, 14 isolates did not carry any element between *mutS* and *mutL*. All other isolates either carried an element identical to MGAS10750 if they were in the MGAS10750-like lineage, or a degraded element identical to MEW427 if they were within the MEW427-like lineage.

Interestingly, although UK isolates were divided between the MEW427-like lineage and MGAS10750-like lineage, North American isolates appeared to be almost exclusively restricted to the MEW427-like lineage. Given that the PHE isolates (UK) [27, 47] were collected at a similar time to the USA ABCs collection (2014–2015) [44], we compared the numbers of isolates present in each lineage: 57/153 (37 %) of PHE isolates were MEW427-like compared to 47/48 (98 %) of ABCs isolates.

## Discussion

Genotype *emm*4 *S. pyogenes* are a major *emm*-type causing disease worldwide [1], with the capacity to cause both self-limiting and potentially life-threatening invasive infection, and a known association with scarlet fever [27–30]. We have identified a lineage within an international *emm*4 population that is characterized by degradation within integrated prophage genomes. Genetic modules pertaining to replication, regulation and lysogeny were chiefly affected, and to a more variable extent, structural genes necessary for the formation of infectious phage particles. Prophage-associated virulence factors were not affected. We confirmed that, at least in the M4_degraded_ isolates in our sample of ten *emm*4, this degradation resulted in prophage immobility.

The lysogenic phage of *S. pyogenes* follow a typically lambdoid genomic architecture, with discrete modules dedicated to specific functions [5, 54, 55]. The most obvious characteristic that we observed in the prophage-encoding regions of the MEW427-like BSAC *emm*4 isolates was the loss of replicative and regulatory modules, as well as deletions within the integrase genes of the prophages Φ427.2 and Φ427.3. From our study, we cannot predict the order in which gene loss occurred, but it seems likely that if a mutation were to occur in the genetic apparatus necessary for excision, selection would presumably act rapidly on those genes remaining that no longer serve a function that is beneficial to the bacterium [56]. As such, the genes or modules remaining raise interesting questions regarding their utility. The retention of prophage-encoded virulence factors is perhaps unsurprising, as multiple lines of scientific investigation have highlighted these genes as conferring a fitness advantage to *S. pyogenes* at the host–pathogen interface [6, 9]. A potential hypothesis is that genomic inactivation of prophage by the bacterium is part of an ongoing process of domestication, whereby the bacterium may retain genes that are useful, particularly toxin genes, while neutralizing the imminent threat of host cell lysis following the induction of the lytic pathway [56].

Our collection of ten *emm*4 isolates could be divided, with the exception of one isolate, into M4_complete_ and M4_degraded_, as the number of genes present within each of the three prophages reflected that of either the complete (MGAS10750) or degraded (MEW427) reference genome prophages. This was based on the full sequence of each prophage region extracted from *de novo* assembly of either short-read or long-read sequence data. For analysis of the wider *emm*4 genomic collection, we used a blast alignment method to estimate prophage composition. We restricted the findings to only full-length genes with ≥99 % identity, meaning only the presence of MGAS10750 prophage genes were detected, and not any truncated genes found in degraded MEW427-like prophages. It was not possible to directly compare prophage sequence because, for the majority of isolates, prophages assembled into multiple contigs. This was either due to other prophage content, and/or variation in sequence and assembly quality. However, from this analysis we could broadly estimate that there was gene loss in all three prophages associated with isolates that clustered with MEW427, but not isolates that clustered with MGAS10750. Further evidence of degradation in the MEW427-lineage came from the fact that all MEW427-lineage isolates carried the same deletion, leading to a predicted non-functional integrase of the *spd3-*prophage, and the majority (96 %) also carried the deletion within the *ssa*-prophage integrase. These integrase deletion mutations were not found in isolates outside of this lineage. We also identified the presence of the Φ427.1 gene *02770*, which had formed following gene loss, in 95 % of MEW427-associated isolates. Therefore, we predict that all isolates belonging to the MEW427-like lineage have at least one immobile and cryptic prophage, and it seems likely that all three prophages would be cryptic in many of these isolates.

It was interesting to note that as well as degradation within the three prophage genomes, we also identified degradation within the chromosomal island SpyCI in our M4_degraded_ BSAC isolates, and in the wider population. Apart from isolates with no SpyCI, all MEW427-lineage isolates had an identical degraded SpyCIM4, like MEW427, and all MGAS10750-lineage isolates had an identical complete SpyCIM4, like MGAS10750. There was one exception, BSAC_bs1802, which had a different SpyCI element entirely. The smaller lineage of 13 isolates also had a different SpyCI element, suggesting these elements can be diverse, even within a single genotype. We expected to find excision of SpyCIM4 from the M4_complete_ lineage BSAC isolates during exponential growth or upon exposure to mitomycin C, as observed for SpyCIM1 in SF370 [12]. Indeed, the excision of SpyCI is reportedly essential for the transcription of *mutL*. However, we could not detect the excision of SpyCIM4 from M4_complete_ BSAC isolates, nor M4_degraded_ BSAC isolates, under either condition tested. Despite this, transcription of both *mutL* and *mutS* were sustained. It seems that in *emm*4, the MMR operon is not regulated by the integration and excision of the element as has been described in other *emm*-types [13], although it is possible that low, undetectable levels of SpyCI excision may occur; thus, permitting transcription of *mutL*. We would expect, however, that this would not be the case for the M4_degraded_ isolates, due to the substantial gene loss within this element, yet *mutL* transcription still occurred. The inability to detect SpyCI in its extra-chromosomal conformation in MGAS10750-like isolates, yet apparently retaining a full complement of associated genes relative to the completed reference genome, may indicate that the element can be induced under conditions not explored in the present study, for example in response to antibiotics [57, 58] or factors produced by the human host *in vivo* [59–62]. It is also possible that due to its stably integrated state, the element was subjected to deletion of surplus genes. In a previous study, wherein the SpyCI element was cured from an SF370-like *emm*1 isolate, this resulted in dramatic changes to global transcription [14]. Retaining a partial or full-length SpyCI in *emm*4 may, therefore, serve a function that extends beyond the DNA MMR operon. Additionally, the gene loss within SpyCI, and potentially the other prophage genomes too, might have a wider influence on the phenotype of the bacterium, through as yet unidentified transcriptional or even post-transcriptional regulatory mechanisms.

An interesting find was the geographical divide, whereby all isolates from the USA collection [44], bar one, were found to be MEW427-like, likely representing degraded prophages, compared to 37 % of UK isolates [27, 47]. This may be related to the over-representation of *emm*4 in the UK associated with scarlet fever [27], although we did not find any association with scarlet fever and *emm*4 lineage within this small dataset with available clinical information; 59 % (16/27) of scarlet fever isolates were M4_complete_. There is a potential that the prophage, either through direct regulation or excision and replication, may have an impact on the expression of prophage-encoded virulence factors, which include the scarlet fever-associated superantigens. It has previously been shown that induction of toxigenic prophage in *S. pyogenes* and *Streptococcus canis* can enhance expression of the cognate virulence factors [59–61, 63]. Similar findings have been described in enterohaemorrhagic *Escherichia coli *, wherein toxigenic prophage induction appears to contribute to the pathogenesis of toxin-mediated disease [58, 64]. It is, therefore, conceivable that the inactivation of prophage in genotype *emm*4 *S. pyogenes* may represent a progression towards a less virulent phenotype, which may be associated with M4_degraded_ isolates, owing to an inability to elicit a gene dosing effect of prophage-encoded virulence factors. It is worth noting, however, that a gene dosing effect does not appear to be necessary in all cases to attain enhanced transcription of these genes [59].

There are other differences separating the two lineages in addition to the prophage degradation, and these may also impact on the behaviour and success of the lineage. A recent study on *emm*4 isolates from Houston, TX, USA, found isolates with a novel chimeric *emm,* formed from fusion with the downstream *enn* gene [36], and chimeric *emm* genes have subsequently been detected in a number of other *emm*-types [65]. The chimeric *emm* was previously found in MEW427 and MEW427-like isolates but not in MGAS10750. We identified the chimeric *emm* in all our M4_degraded_ BSAC isolates, but not in our M4_complete_ BSAC isolates. The chimeric *emm* gene could influence the phenotype of the MEW427-like isolates in addition to the prophage degradation. Gubbins analysis of our BSAC *emm*4 also identified a region of recombination surrounding MGAS10750 genes Spy485–488, and these are absent in MEW427 and MEW427-like isolates. The function of these genes is not known, although Spy485 is annotated as microcin C7 self-immunity protein MccF. These genes are found in isolates belonging to some genotypes, but absent in others, indicating their presence in the *S. pyogenes* genome is variable.

It is not clear what other impacts prophages have on *S. pyogenes* during infection, beyond the transfer and expression of associated virulence factors. The cryptic prophage of *E. coli* may bolster the success of lysogens in challenging environments, and in response to certain stimuli, such as growth in nutrient poor conditions, extremes of pH, oxidative stressors and antibiotics [66], and perhaps this is the case for M4_degraded_ *emm*4. It is also conceivable that by harbouring neutralized prophage that cannot induce lysis of the host bacterium, M4_degraded_ are better able to resist certain challenges encountered during growth at non-sterile sites such as the human nasopharynx, the primary anatomical niche of *S. pyogenes. Streptococcus pneumoniae*, another bacterium also often present in the human nasopharynx, may exploit the lysogenic nature of its competitors, specifically by secreting hydrogen peroxide and triggering prophage induction and lysis in *Staphylococcus aureus* lysogens [67]. Resilience to this form of competitive insult might, therefore, confer an advantage to M4_degraded_ during infection. Human pharyngeal cells alone appear to trigger prophage induction and particle formation in *S. pyogenes* [59, 60] and, thus, their inactivation may confer a possible survival advantage *in vivo*. Prophage also appear to play a role in the complex and elusive competence pathway(s) of *S. pyogenes *. Indeed, the prophage-borne ‘paratox’, which is often contiguous with genes pertaining to virulence in streptococcal phage, seemingly interferes with interactions between proteins involved in the mediation of transformation and recombination; thus, dramatically reducing competence [68]. These hypotheses represent exciting targets for future study, and may enhance current understandings of the infection biology of *S. pyogenes* and the role of prophages at the host–pathogen interface.

Full *de novo* assembly of integrated prophages is notoriously difficult due to the homologous and mosaic nature of these genomes. We were unable to fully assemble these regions within single contigs in isolates where at least one other prophage was present due to the homology between prophages. Long-read sequencing of four isolates produced complete genome sequences that led to the identification of additional prophages within five of the BSAC *emm*4 genomes. BSAC_bs1802 was unusual in that it not only carried a different *speC/spd1* prophage and a unique SpyCIM4 element, but also carried a prophage associated with the DNase *sdn,* which was predicted to be complete. Four other BSAC isolates, BSAC_bs192, BSAC_bs400, BSAC_bs472 and BSAC_bs1388, each had one additional prophage. The prophages in these isolates were closely related to each other, but only Φ192.1 was associated with *speK/sla,* and Φ192.1 and Φ427.2 had a different integration site to Φ400.1 and Φ1388.1. This suggests that these prophages are less stable in the population than the Φ10750.1/427.1, Φ17050.2/427.2 and Φ107050.3/427.3 prophages. The presence of structural, replication and regulation regions suggested that they were not degraded, even within M4_degraded_ strains BSAC_bs192 and BSAC_bs472, compared to M4_complete_ BSAC_bs1388. The relatively high level of homology shared between these additional prophages and canonical *emm*4 prophages (80–100 % homology between ~20 % of prophages genes), introduced breaks in the short-read sequence *de novo* assembly, and long-read sequencing was required to determine exact composition. This need for long-read sequencing, unfortunately, hampers current work into prophage genetics within the streptococcal population, about which we know little but, given our observations with just ten isolates, could be highly variable. There has been some evidence for prophage-mediated genome rearrangements [69] and the hybridization of different prophages giving rise to increased toxin expression [19]. It also seems likely that prophage degradation exists in other genotypes, as well as *emm*4.

There is great potential for prophages to drive evolutionary changes within the streptococcal population. Their mechanisms of impact may range from the simple transfer of virulence factors, through to local- and genome-wide transcriptional control, and even direct phenotypic influences, through induction, particle formation and host cell lysis. Therefore, we need to expand our knowledge and research in this area to fully appreciate the role prophages play in the population of streptococci, and indeed other bacterial species.

## Supplementary Data

Supplementary material 1

Supplementary material 2
