# Supplementary material for: Cryptic prophages within a Streptococcus pyogenes genotype emm4 lineage
Source: Microb Genom. 2020 Nov 27;7(1):mgen000482. doi: 10.1099/mgen.0.000482 (PMC8115907; doi:10.1099/mgen.0.000482)
Supplement: Supplementary material 1 [file mgen-7-482-s001.pdf]

Supplementary Information for:

Cryptic prophages within a *Streptococcus pyogenes* genotype *emm*4 lineage

Alex Remmington, Samuel Haywood, Julia Edgar, Luke R. Green, Thushan de Silva, Claire E. Turner.

Supplementary Tables

Supplementary Table 1. Completed genomes.

Isolate	Accession No.	Total length	Prophages
BSAC_bs192	CP061134	1,865,571 bp	Φ427.1 (*speC/spd1*)<br>Φ427.2 (*spd3*)<br>Φ427.3 (*ssa*)<br>Φ192.1 (*speK/sla*)
BSAC_bs472	CP061133	1,859,391 bp	Φ427.1 (*speC/spd1*)<br>Φ427.2 (*spd3*)<br>Φ427.3 (*ssa*)<br>Φ472.1 (none)
BSAC_bs1388	CP061132	1,932,413 bp	Φ10750.1 (*speC/spd1*)<br>Φ10750.2 (*spd3*)<br>Φ10750.3 (*ssa*)<br>Φ1388.1 (none)
BSAC_bs1802	CP061131	1,909,430 bp	Φ1802.1 (*speC/spd1*)<br>Φ427.2 (*spd3*)<br>Φ10750.3 (*ssa*)<br>Φ1802.2 (*sdn*)

Supplementary Table 3. Primers used in the study

**Primer**	**Sequence (5'-3')**	**Function**
10750.1 A	GCATCCAGACTATTCCATTC	Φ10750.1/Φ427.1 induction
10750.1 B	CGTATGATGTTCAATCTAGGATAG	
10750.1 C	TGCGTCAACAGTTATTGTCG	
10750.1 D	ACATTAGCCTCGTTCACGC	
10750.2 A	ATCAACTAAGGCAGCTTCTG	Φ10750.2/Φ427.1 induction
10750.2 B	CGGAACTCTTGACTACACCTC	
10750.2 C	AACAAACCTTGCCAAGTACG	
10750.2 D	CCATCTCTGTAACAGTCAAATG	
10750.3 A	CCAATCAAGAAGGCTGTAATG	Φ10750.3/Φ427.1 induction
10750.3 B	GCACCTGGAGCAATATTTG	
10750.3 C	TACAGAAGGATATCGTAACGGG	
10750.3 D	TTGCAAGTCGTCTCATTCAAG	
SpyCIM4 A	CGAGAACTTCCGGTAATTC	SpyCIM4 induction
SpyCIM4 B	CGAATATCAGCATGACTTTG	
SpyCIM4 C	AGCATCCAAGACCAATGG	
SpyCIM4 D	CTTCAAGCAATGACAACCC	
MutL_F	GTCTCAATTTCCCCACCAGTAG	*mutL* transcription
MutL_R	CAAATTGCAGCTGGTGAAG	
MutS_F	CTTGAAGCGGGGTCATATTC	*mutS* transcription
MutS_R	GTTGGTGCTAAGACCATATTTGC	

Supplementary Figures

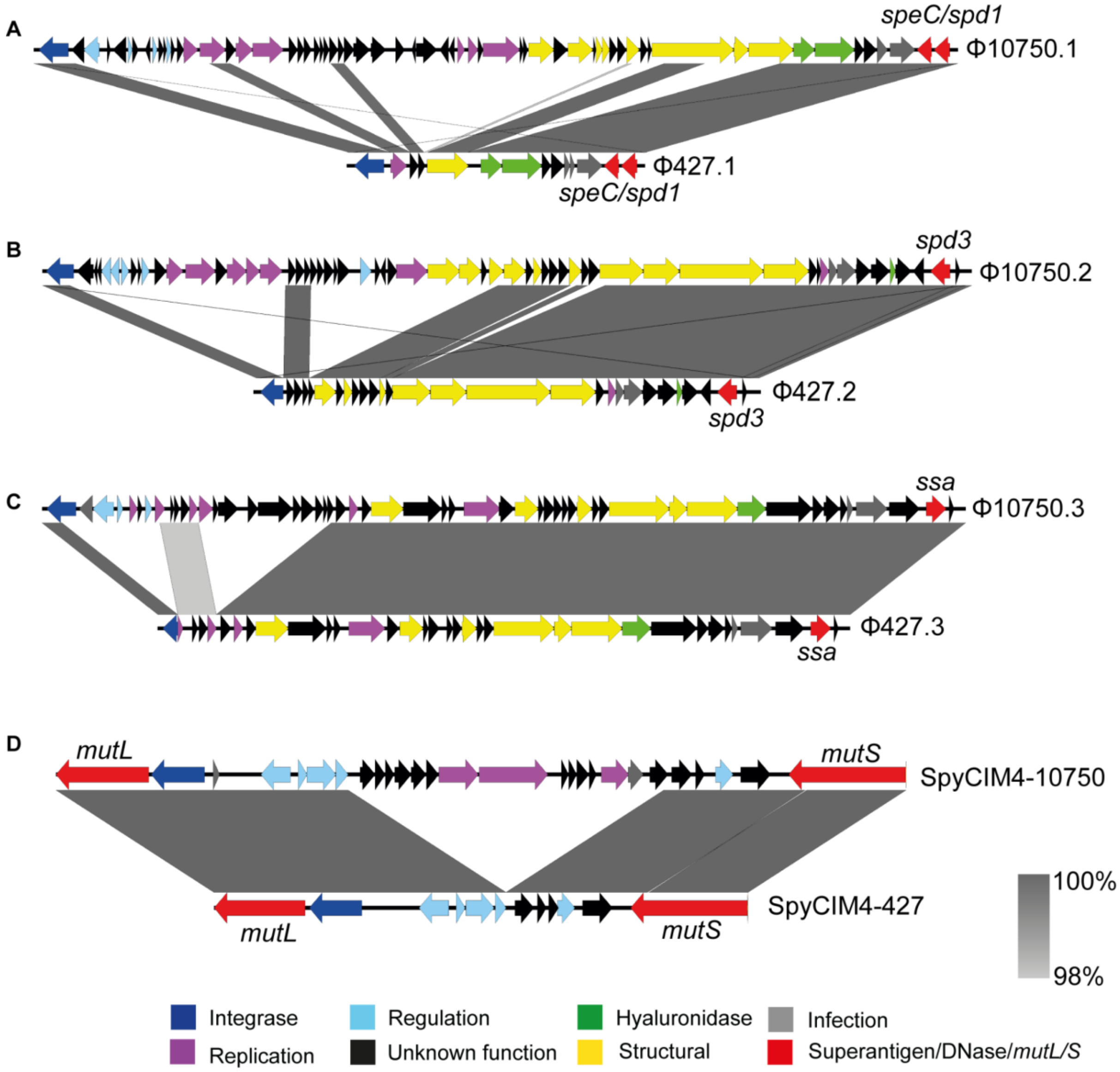

Supplementary Figure 1. Gene loss within the prophage and SpyCI regions of MEW427 compared to MGAS10750. The regions of each MGAS10750 prophage and SpyCI element were extracted from the genome and compared to corresponding regions in MEW427 genome using EasyFig (Sullivan et al. 2011). All three prophages (**A-C**) and the SpyCI (**D**) of MEW427 were much shorter than in MGAS10750, relating to loss of sequence and consequently loss of genes. Colours represent predicted gene function according to the key. Grey shade indicates sequence identity determined by BLAST.

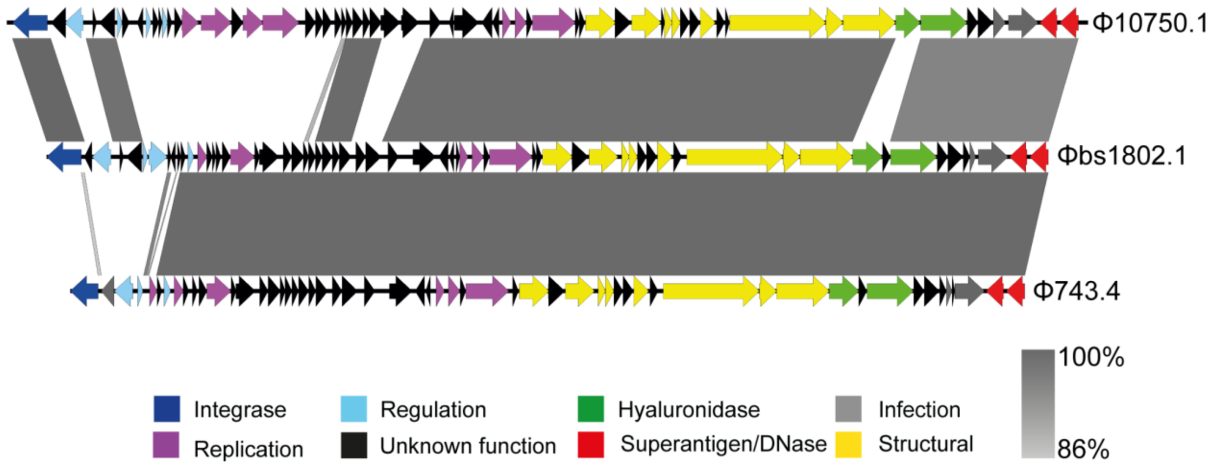

Supplementary Figure 2. Comparison of *speC/spd1* associated prophages. The prophage in BSAC_bs1802 associated with *speC/spd1* (Φ1802.1) showed some homology to Φ10750.1 but varied at the integrase end. Φbs1802.1 was more closely related to Φ743.4 found in *emm*87 isolate NGAS743 (gene loci: DI45_06730-DI45_07035, Genbank accession: CP007560.1) (Athey *et al.* 2016) although still differed at the integrase end. Colours represent predicted gene function according to the key. Grey shade indicates sequence identity determined by BLAST. Figure constructed using EasyFig (Sullivan et al. 2011).

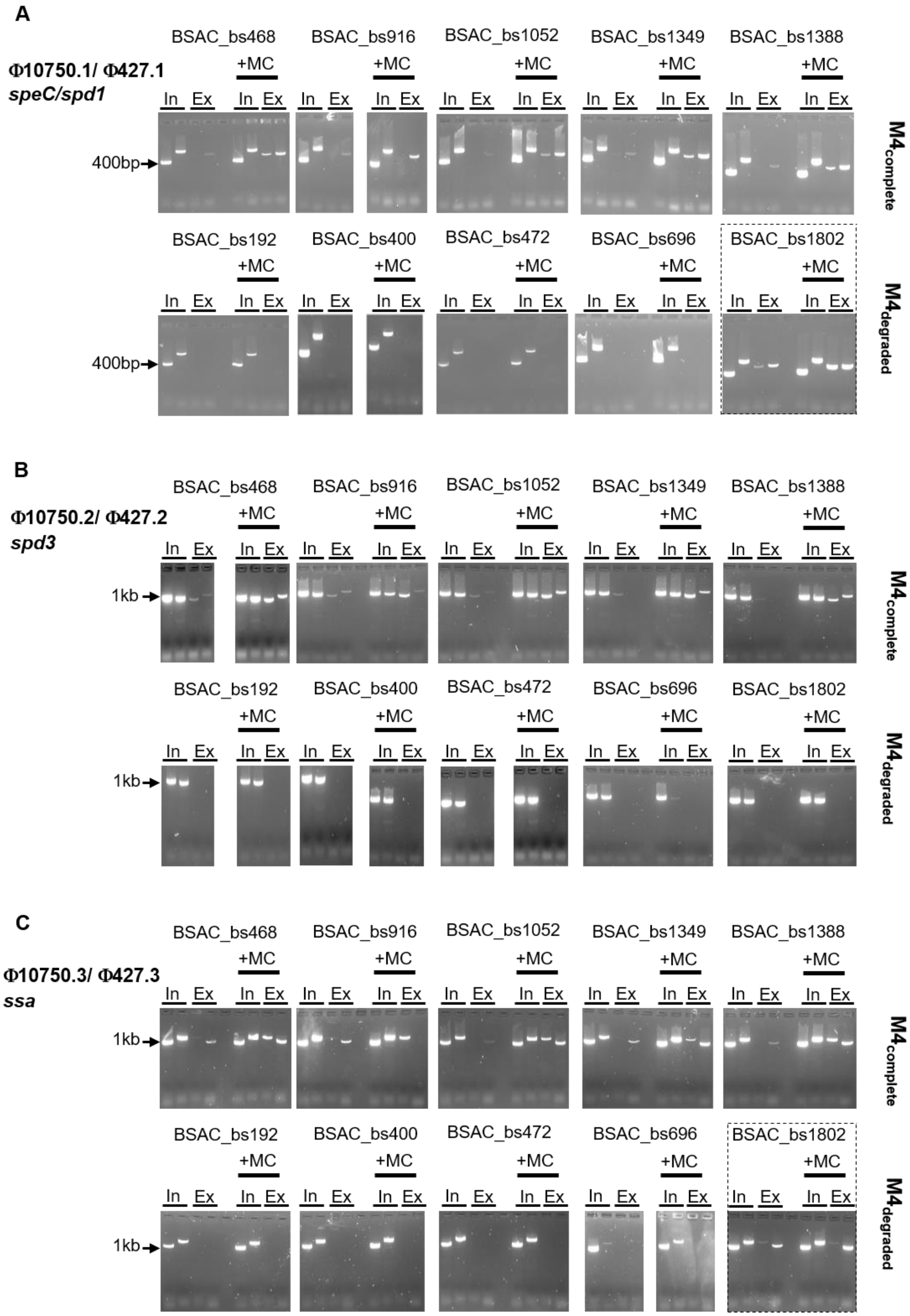

A
Φ10750.1/ Φ427.1
speC/spd1
BSAC_bs468
BSAC_bs916
BSAC_bs1052
BSAC_bs1349
BSAC_bs1388
+MC
In
Ex
400bp
M4complete
BSAC_bs192
BSAC_bs400
BSAC_bs472
BSAC_bs696
BSAC_bs1802
M4degraded
B
Φ10750.2/ Φ427.2
spd3
1kb
C
Φ10750.3/ Φ427.3
ssa

Supplementary Figure 3. Phage induction in $M4_{complete}$ isolates but not $M4_{degraded}$ isolates. Both integrated prophage, as indicated by bands in the two 'In' lanes, and excised prophage, as indicated by bands in the two 'Ex' lanes (primer pairs A+D and C+B) were detected in all five $M4_{complete}$ isolates and for all three prophages; (**A**) Φ10750.1/Φ427.1, (**B**) Φ10750.2/Φ427.2, (**C**) Φ10750.3/Φ427.3. Excision was enhanced by the addition of mitomycin C (+MC). No excision was detected for all three prophages in all of the four $M4_{degraded}$ isolates, as indicated by bands only in the 'In' lanes but not the 'Ex' lanes, even with mitomycin C. BSAC_bs1802 groups with $M4_{degraded}$ but carried a different full-length *speC*/*spd1* prophage, which was inducible and is highlighted with a dotted box (**A**). The Φ10750.3/ Φ427.3 *ssa* prophage is also inducible in this isolate compared to the other $M4_{degraded}$ isolates (dotted box, **C**). Isolates for which PCR products from with and without MC were not run in parallel on the same agarose gel are presented as separate but contiguous panels. Arrows represent molecular weight.

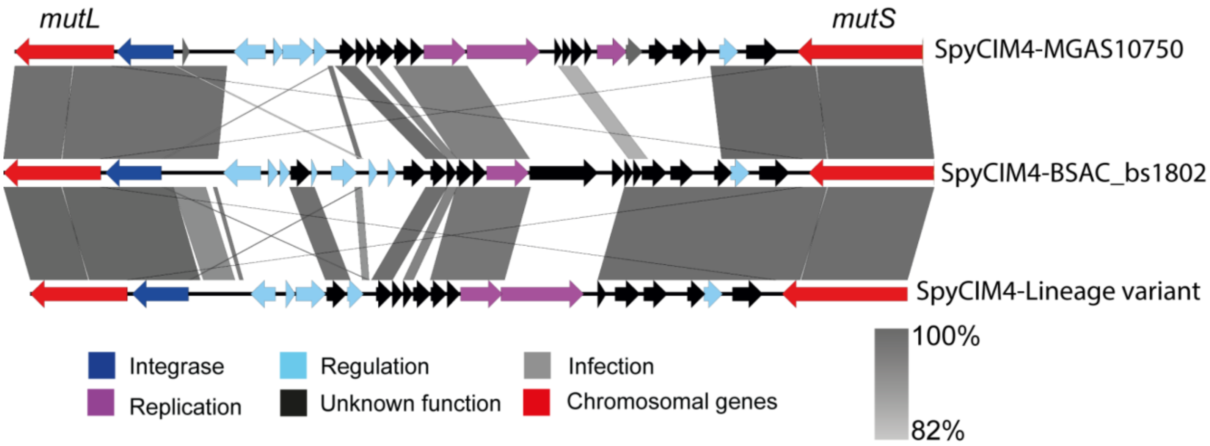

Supplementary Figure 4. SpyCIM4 elements identified within the population. Apart from the SpyCIM4-MGAS10750 and the degraded SpyCIM4-MEW427, two other variants of SpyCIM4 were identified in the population. One was unique to BSAC_bs1802 (SpyCIM4-BSAC_bs1802) and the other was found in the small 13 isolate lineage (indicated by a dotted line in Figure 5) (SpyCIM4-Lineage variant). Colours represent predicted gene function according to the key. Grey shade indicates sequence identity determined by BLAST. Figure constructed using EasyFig (Sullivan et al. 2011).

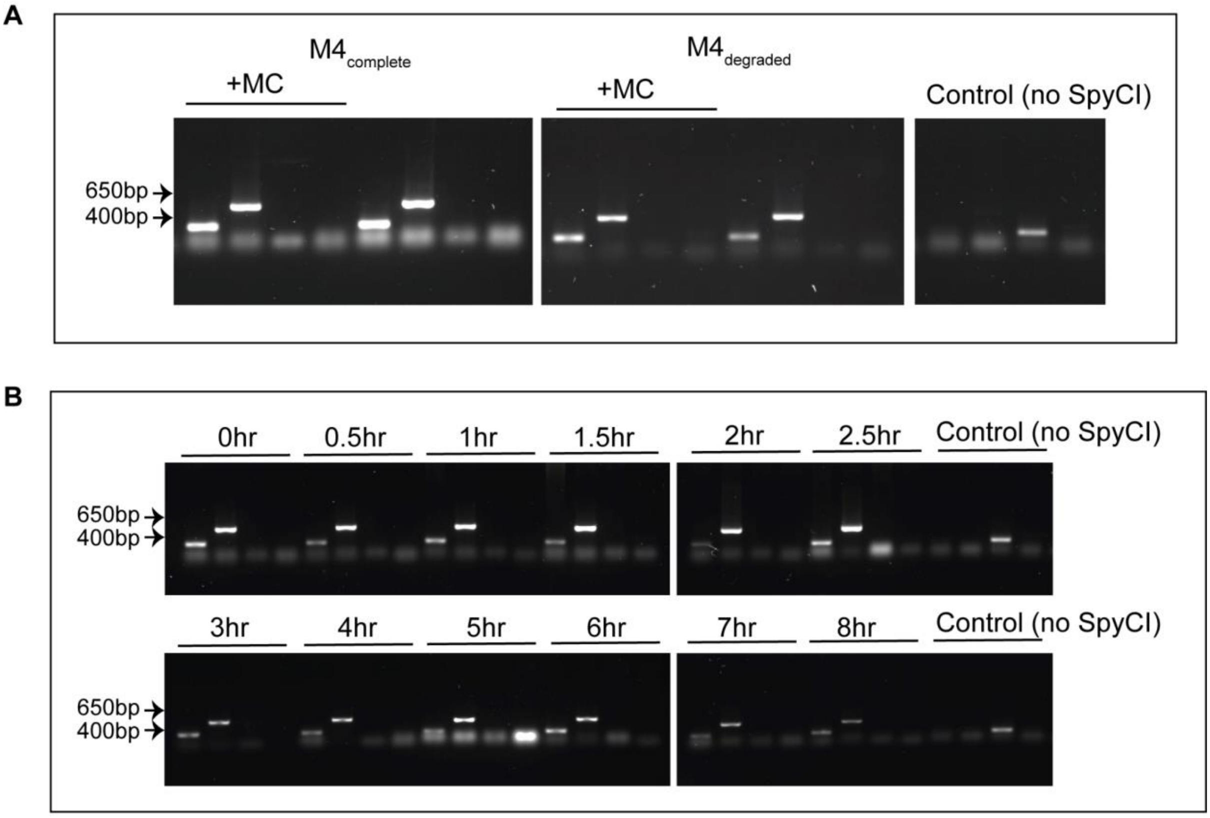

Supplementary Figure 5. SpyCIM4 is not induced in any of the *emm*4 BSAC isolates. (A) Primers were designed to detect integrated and excised SpyCI in genotype *emm*4 isolates in response to induction with mitomycin C. Only integrated SpyCI was detected (bands in first two lanes per sample) in both $M4_{complete}$ and $M4_{degraded}$ isolates, and no evidence of excised SpyCI (third and fourth lanes per sample), even in the presence of mitomycin C (+MC). **(B)** To determine if the *emm*4 SpyCI would excise at specific stages during liquid growth of an $M4_{complete}$ isolate, samples were taken at 30-minute intervals from 0-3 hours, then at 60-minute intervals thereafter, for a further 5 hours. Excised SpyCI was not detected at any point. A control DNA sample was also used in each experiment extracted from *emm*89 isolate H293; this isolate has no SpyCI element and therefore only a band in third lane can be detected. Arrows indicate size (base pairs) positions from the marker.

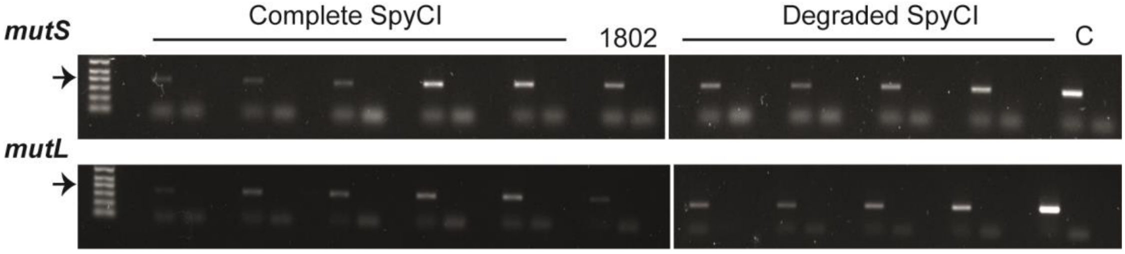

Supplementary Figure 6. Expression of both *mutL* and *mutS* detected in both M4$_{complete}$- and M4$_{degraded}$ type *emm*4 isolates. Bacterial RNA was extracted from M4$_{complete}$- and M4$_{degraded}$ isolates at 3 hours of growth (early log) and converted to cDNA by reverse transcription (RT). 100ng of cDNA was used to detect *mutL* or *mutS* transcript by PCR. Each isolate is represented by two lanes; the first is the RT positive sample (i.e. containing cDNA), the second is a negative control whereby RT was excluded. Transcription of *mutS* (top gel) and *mutL* (bottom gel) was detected in all five BSAC M4$_{complete}$ as well as all four BSAC M4$_{degraded}$ isolates. Transcripts for *mutS* and *mutL* were also detected in BSAC_bs1802 which carries a different SpyCI element to the other *emm*4 isolates. C; DNA from control SpyCI-negative H293 in the first lane, no-DNA sample in the second lane. Arrows indicate 400bp on the 1kb Plus Ladder (Invitrogen).

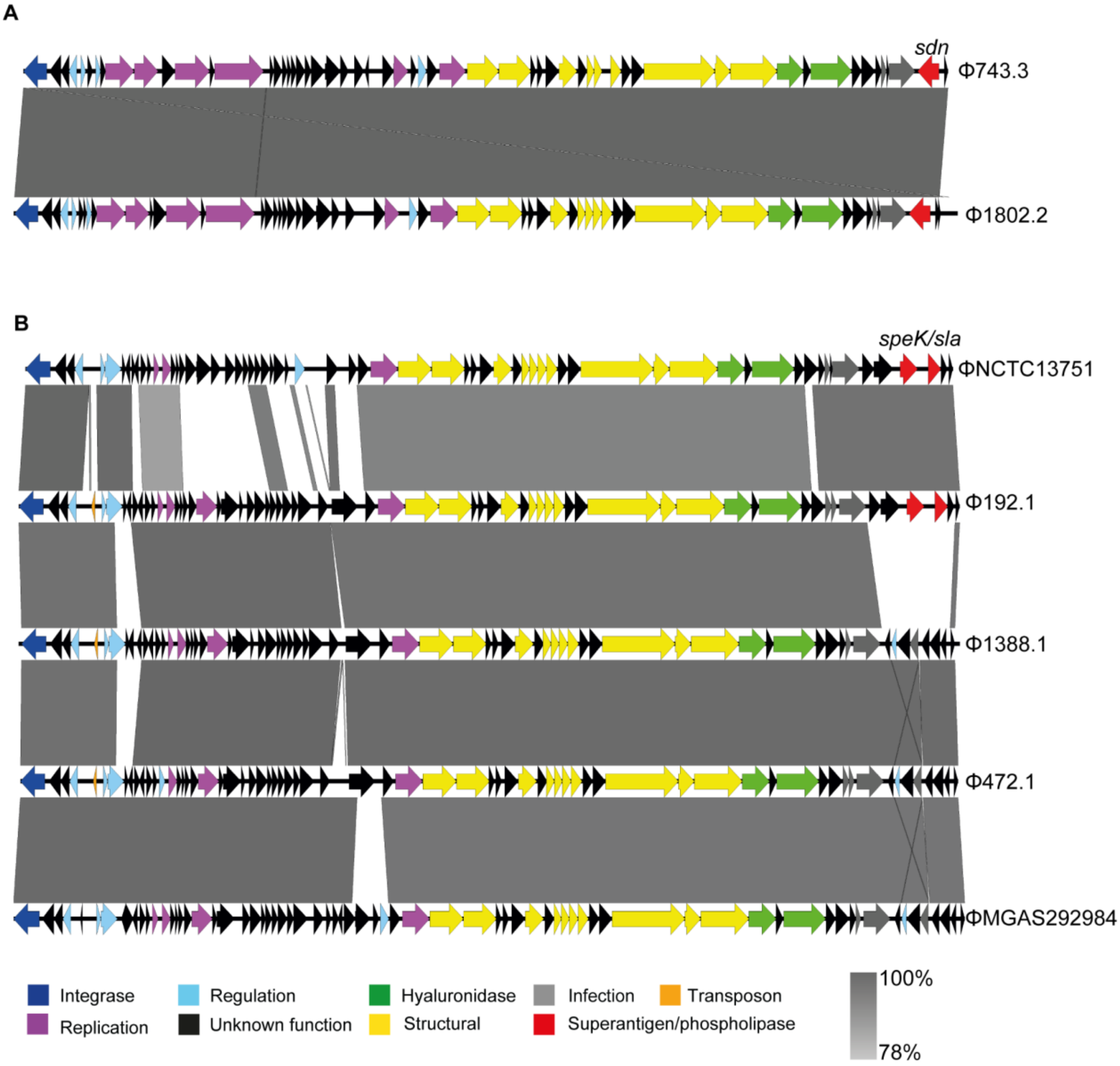

Supplementary Figure 7. Comparison of additional prophages found in the BSAC isolate genomes. (A) An additional prophage was found in BSAC_bs1802 (Φ1802.2) associated with the DNase *sdn*. This phage was almost identical to Φ743.3 found in *emm*87 isolate NGAS743 (gene loci: DI45_06355-DI45_06665, Genbank accession: CP007560.1 (Athey *et al.* 2016) and was found to be integrated in an equivalent position. (**B**) BSAC_bs192 was found to carry a prophage associated with the superantigen *speK* and the phospholipase *sla* (Φ192.1). This prophage shared closest homology with a prophage in the genome of NCTC13751 (gene loci: NCTC13751_1363-NCTC13751_1427, Genbank accession: LS483437.1). However, there was also a high level of homology between Φ192.1 and phages Φ1388.1 and Φ472.1 found in

BSAC_bs1388 and BSAC_bs472, respectively. Φ1388.1 and Φ472.1 did not carried *speK/sla* or any known virulence factors, and Φ1388.1 was integrated into a different site to Φ192.1 and Φ472.1, that shared integration sites. A prophage within the genome of isolate MGAS29284 (gene loci: DZ066_3390-DZ066_3790, Genbank accession: CP031619), which does not carry *speK*/*sla* or any known virulence factors, shared the closest homology with Φ192.1, Φ1388.1 and Φ472.1 and integrated in an equivalent position to Φ192.1/Φ472.1. Colours represent predicted gene function according to the key. Grey shade indicates sequence identity determined by BLAST. Figure constructed using EasyFig (Sullivan et al. 2011).

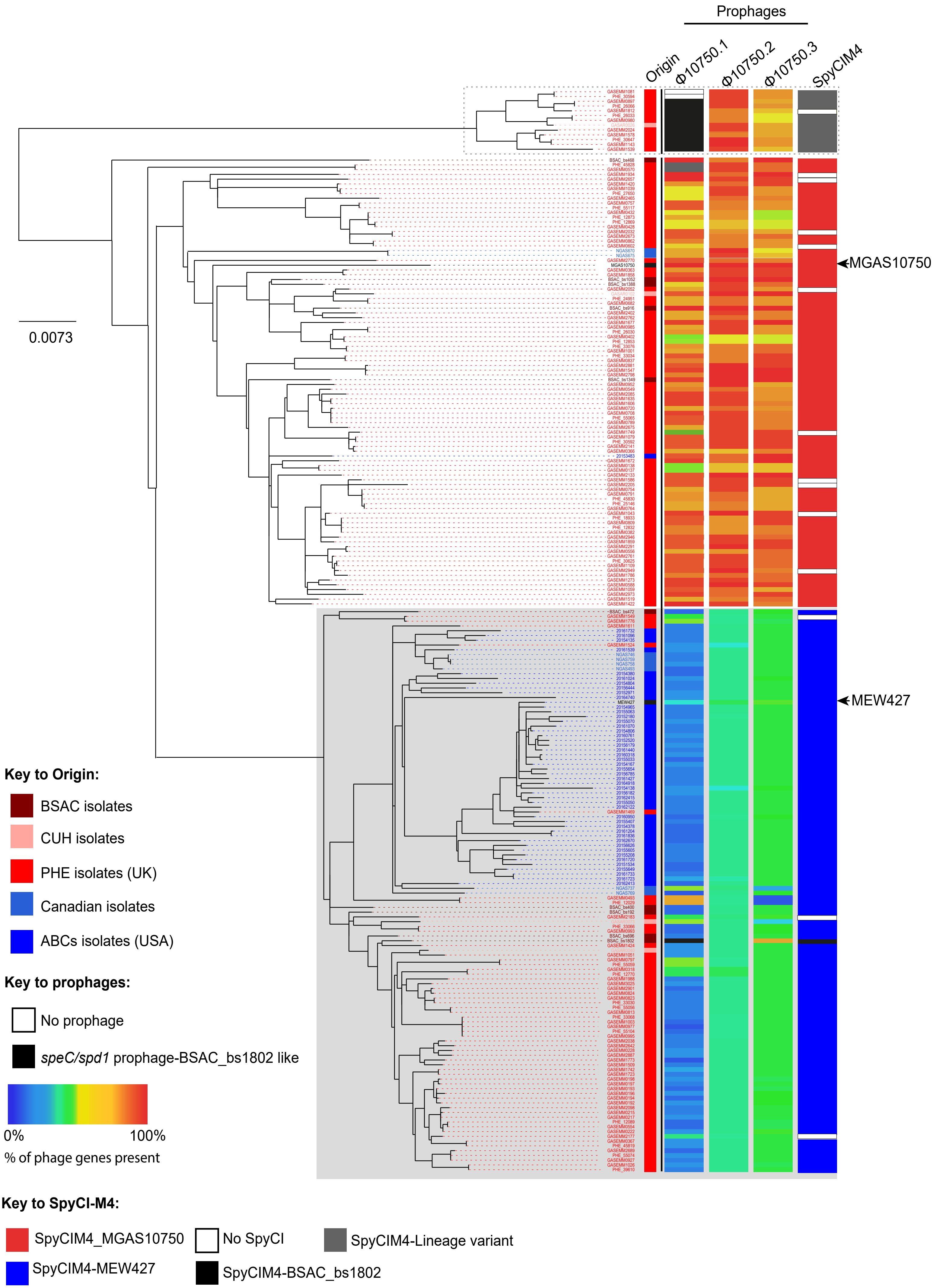
Prophages
Origin
Φ10750.1
Φ10750.2
Φ10750.3
SpyCIM4
MGAS10750
MEW427
0.0073
Key to Origin:
BSAC isolates
CUH isolates
PHE isolates (UK)
Canadian isolates
ABCs isolates (USA)
Key to prophages:
No prophage
speC/spd1 prophage-BSAC_bs1802 like
0%
100%
% of phage genes present
Key to SpyCI-M4:
SpyCIM4_MGAS10750
No SpyCI
SpyCIM4-Lineage variant
SpyCIM4-MEW427
SpyCIM4-BSAC_bs1802

Supplementary Figure 8. Lineages broadly associated with levels of prophage gene presence. Mid-rooted phylogenetic tree constructed from core SNPs obtained after mapping short-read sequence data from 223 isolates as well as the reference genome MEW427 to MGAS10750 (as Figure 5). Lineages are defined as in Figure 5; MGAS10750-like (no shading), MEW427-like (grey shaded), separate 13 isolate lineage (dotted-line). The percentage (%) of genes present in the *de novo* assembled genome for each isolate was calculated by BLAST analysis for each of the three MGAS10750 prophages (Φ10750.1-3) and indicated by colour scale (key indicated). Two isolates within the MGAS10750 lineage did not have the Φ10750.1 prophage as indicated by a white box in the first prophage column. The identification of SpyCIM4 regions was determined by sequence alignment and the majority were found to have either SpyCIM4 as MGAS10750 (red) or as MEW427 (blue). BSAC_bs1802 had a unique SpyCIM4 (black) and the isolates belonging to the lineage highlighted by a dotted line had a fourth type of SpyCIM4 (Lineage variant, grey) (see Supplementary Figure 3). Isolates names are given for each branch and colour coded as in the first column (Origin) based on collection; BSAC isolates (n=10), CUH (Cambridgeshire n=4 (Turner *et al.* 2017)), PHE (UK, n=153 (Chalker *et al.* 2017; Kapatai *et al.*, 2017)), Canadian (n=8 (Athey *et al.* 2014, Athey *et al*. 2016)), ABCs (USA, n=48 (Chochua *et al.*, 2017)). Scale bar represents substitutions per site.
